# Genome-Wide Identification and Expression Analysis of Hexokinase Gene Family Under Abiotic Stress in Tomato

**DOI:** 10.3390/plants14030441

**Published:** 2025-02-03

**Authors:** Jing Li, Xiong Yao, Jianling Zhang, Maoyu Li, Qiaoli Xie, Yingwu Yang, Guoping Chen, Xianwei Zhang, Zongli Hu

**Affiliations:** 1 Laboratory of Molecular Biology of Tomato, Bioengineering College, Chongqing University, Room 523-1, Campus B, 174 Shapingba Main Street, Chongqing 400030, China; micy180605@163.com (J.L.); qiaolixie@cqu.edu.cn (Q.X.); yangyinwu@cqu.edu.cn (Y.Y.); chenguoping@cqu.edu.cn (G.C.); 2 Chongqing Academy of Agricultural Sciences, Chongqing 401329, China; yuyourice@cqaas.cn; 3 Laboratory of Plant Germplasm Resources Innovation and Utilization, College of Agriculture and Biology, Liaocheng University, Liaocheng 252000, China; zhangjianling0520@126.com; 4 Chongqing Seed Station, Chongqing 401121, China; m18523046893_1@163.com

**Keywords:** tomato, hexokinase, molecular characterization, expression pattern, abiotic stress

## Abstract

In plants, hexokinase (HXK) is a kind of bifunctional enzyme involved in sugar metabolism and sugar signal transduction that plays important roles in plant growth and development and stress response. Some HXK genes without a phosphorylation function have been found in Arabidopsis, tobacco, etc., but these genes have not been identified in tomato. Therefore, further genome-wide systematic identification and characterization is necessary for tomato HXK genes. In this study, six HXK genes were identified from the tomato genome distributed across six different chromosomes, named *SlHXK1-6*. Gene structure analysis showed that the *SlHXK* genes contain the same number of introns and exons. Gene duplication and collinearity analysis revealed two pairs of tandem repeats among *SlHXKs*, and a higher collinearity between tomatoes and potatoes were found. Response elements associated with phytohormones, abiotic stresses, and growth and development were identified in the promoter sequences of *SlHXKs*. Quantitative real-time PCR (qRT-PCR) results further indicated the potential role of *SlHXKs* in tomato development and stress responses. The expression levels of most *SlHXKs* were significantly induced by abiotic stress, hormone, and sugar solution treatments. In particular, the expression of *SlHXK1* was significantly induced by various treatments. Functional complementation experiments were performed using HXK-deficient yeast strain YSH7.4-3C (*hxk1*, *hxk2*, and *glk1*), and the results showed that SlHXK5 and SlHXK6 were unable to phosphorylate glucose and fructose in yeast. In conclusion, these results provide valuable foundations for further exploring the sugar metabolism and sugar signal transduction mechanisms of HXK and the functions of *SlHXK* genes in various abiotic stresses, and some *SlHXKs* may be key genes for enhancing plants’ tolerance to abiotic stresses.

## 1. Introduction

In plants, sucrose is the main carbohydrate produced through photosynthesis. It can be metabolized in the photosynthetic leaves or transported from the leaves to the non-photosynthetic sink tissues (e.g., flowers, seeds, roots, and stems) through phloem [1]. Sucrose can be stored or metabolized into hexoses (fructose and glucose) after being transported to the sinking tissue [2]. Glucose and fructose need to be phosphorylated before entering the metabolic pathway [3]. In plants, hexokinase is the only enzyme that can phosphorylate glucose [4]. Glucose is phosphorylated by hexokinase (HXK) and then enters the glycolysis pathway to provide energy and some important intermediate metabolites for plant growth and development [5]. Hexokinase is a dual-function enzyme that not only has catalytic activity but also acts as a glucose sensor to control gene expression, hormone interactions, and the regulation of plant growth [3,6].

To date, plant HXK gene family members have been identified in many species, such as 6 in *Arabidopsis* [7], 9 in *Nicotiana tabacum* [8], 10 in rice [9], 9 in maize [10], 10 in pear [11], 19 in *Brassica napus* [12], 12 in moso bamboo [13], 7 in cassava [14], 17 in cotton [15], 6 in poplar [16], 4 in *Jatropha curcas* [17], and 4 in *Camellia sinensis* [18]. Previous studies showed that the HXKs are classified into two main groups (type A and type B) and two minor types (type C and type D) based on the N-terminal amino acid sequence [19]. The type B HXKs generally have a hydrophobic membrane anchor domain associated with mitochondria, such as AtHXK1 and 2 and AtHKL1, 2, and 3 from *Arabidopsis* [7], OsHXK3, 5, 6, 9, and 10 from rice [9], and ZmHXK3a, 3b, 4, 5, 6, 9, and 10 from maize [10]. However, some type B HXKs may also be localized in the nucleus due to the presence of nuclear localization signals near their transmembrane domains [20]. Related studies have shown that type B HXKs act as the glucose sensors in primary and secondary metabolism of plants, programmed cell death, and plant growth and development [6,21,22,23,24]. Unlike type B, the type A HXKs (e.g., AtHXK3, NtHXK2, and OsHXK4) contain a chloroplast transit peptide at the N-terminal [7,8,9]. Type C HXKs, such as OsHXK1, 7, and 9 in rice, lack membrane-anchored domains and chloroplast transit peptides and are localized in the nucleus and the cytoplasm [25]. At present, they are only found in monocotyledonous plants and *Physcomitrella patens* [25,26]. In addition, type D HXKs are generally found in gymnosperms, lycophytes, and bryophytes, and they may lack conserved peptides [26].

At present, some studies have shown that HXKs are involved in a variety of stress responses, including biotic stress and abiotic stress [27,28,29]. For example, under low temperature and salt stress, the expression of *AtHXK3* and *AtHXKL1* was significantly inhibited, while the expression of *AtHXK2* was significantly induced in *Arabidopsis thaliana* [30,31]. And, the transcripts of *AtHXKL3* were significantly increased under salt, drought, high-temperature, and low-temperature stresses [31]. In addition, overexpression of *OsHXK1* results in enhanced resistance of rice leaves to virus infection [27]. In apple, overexpression of *MdHXK1* enhanced resistance to the fungal pathogens [28]. In a recent study, it was shown that PdGNC, a GATA transcription factor, regulates stomatal aperture and affects water use efficiency and drought tolerance in *Populus* because HXK activation promotes the accumulation of NO and H_2_O_2_ in guard cells [29]. A large number of genetic, molecular biological, biochemical, and cytological analyses have shown that the glucose signal pathways mediated by HXKs are closely related to a variety of plant hormones, including auxin, gibberellin, ethylene, cytokinin salicylic acid, and abscisic acid [32,33]. In *Arabidopsis*, AtHXK1 was involved in the regulation of SA (salicylic acid)-dependent PCD (programmed cell death), leading to the formation of light-dependent lesions in plant leaves [34]. Moreover, *AtHXK1* was able to promote the degradation of EIN3 and EIL1 in the nucleus, and it can also affect ABA2 in the ABA synthesis pathway and ABI4 and ABI5 in the ABA signaling pathway [35,36]. In addition, *gin2-1*, an AtHXK1 mutant, also exhibited increased sensitivity to cytokinin and reduced sensitivity to auxin [21].

Tomato has become one of the most economically valuable vegetable crops, and it is considered an important model plant for studying plant growth and fruit development. As a bifunctional enzyme, hexokinase not only has catalytic activity and participates in sugar metabolism but also acts as a glucose receptor and mediates sugar signal transduction, which plays important roles in plant growth and development and stress response [3,6]. Previously, we investigated the function of the *SlHXK1* gene and found that it plays important roles in the growth and development of tomato [37,38]. In addition, some HXK genes without a phosphorylation function were found in *Arabidopsis*, rice, etc., which can act as a glucose sensor to control gene expression, hormone interactions, and the regulation of plant growth [39,40]. Although previous studies have identified several members of the tomato HXK family, no HXK gene that cannot phosphorylate glucose and fructose has been found in tomato [41,42,43]. We hypothesized that there are more HXK genes in tomato. Therefore, a systematic and in-depth genome-wide identification of HXK genes in tomato is needed to identify more HXK genes, laying a foundation for further research on the role of HXK in tomato growth and development and stress response.

In this work, we identified and characterized SlHXKs through the investigation of physicochemical properties of the proteins, phylogenetic relationships, collinearity, exon-intron structures, chromosome distribution, and motif analysis. The spatiotemporal expression of *SlHXKs* was analyzed through qRT-PCR in different tissues and at different developmental stages in tomato. Moreover, qRT-PCR was also used to detect the transcriptional level of *SlHXKs* in various abiotic stresses, sugars, and phytohormones. Finally, the HXK-deficient yeast strain YSH7.4-3C was used to explore the HXK activity of SlHXKs through functional complementation experiments. Taken together, these results provide a theoretical basis for further studying the function of *SlHXKs* in tomato growth and development and stress acclimation.

## 2. Results

### 2.1. Identification and Characterization of SlHXK Genes in Tomato

In this research, a total of six *SlHXK* genes were identified. In addition to the four genes (*SlHXK1*-*SlHXK4*) previously identified, the remaining two genes were named *SlHXK5* and *SlHXK6* according to their positions on the tomato chromosome (Figure S1). The gene ID, gene and mRNA length, open reading frame (ORF), amino acid length, isoelectric point (pI), molecular weight (MW), protein stability, hydrophilic–hydrophobic amino acids, transit peptide (TP), transmembrane domain, and subcellular locations of six SlHXK proteins were analyzed (Table 1). The length of the six *SlHXK* genes ranged from 3808 to 5331 bp, and the length of the mRNAs ranged from 1765 to 1862 bp. The open reading frame’s (ORF) length ranged from 1476 to 1536 bp. The amino acids ranged from 491 (SlHXK5) to 511 (SlHXK6). The molecular weight (MW) of SlHXKs ranged from 53.75 kDa (SlHXK2) to 55.50 kDa (SlHXK6). The theoretical isoelectric points of six *SlHXKs* were between 5.52 and 6.26. All SlHXKs were found to be hydrophilic proteins through the grand average of the hydropathicity analysis. According to the analysis of the instability index, three SlHXK proteins (SlHXK1-3) with instability indexes less than 40.0 were stable proteins, while three SlHXK proteins (SlHXK4-6) with instability indexes greater than 40.0 were unstable proteins. More detailed physicochemical properties of SlHXKs are listed in Table 1.

### 2.2. Phylogenetic Relationships and Multiple Alignments

**Figure 1:**
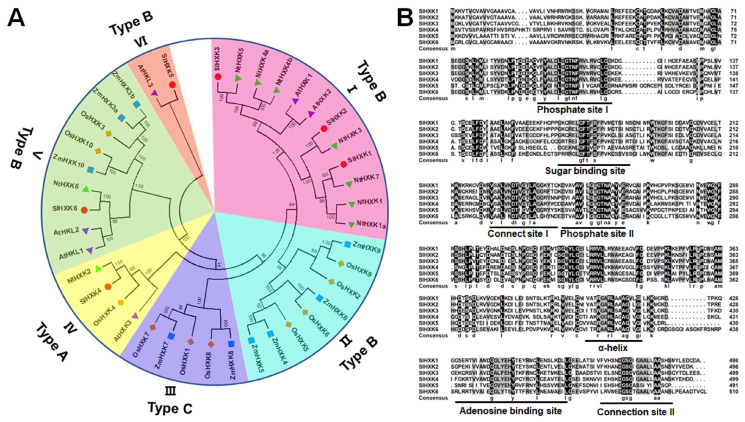
Phylogenetic analysis and sequence analysis of HXK family genes. (**A**). Phylogenetic analysis of HXK proteins from *Solanum lycopersicum*, *Arabidopsis thaliana*, *Nicotiana tabacum*, *Oryza sativa*, and *Zea mays*. Accession numbers are as follows: SlHXK1 (NP_001233957.1), SlHXK2 (NP_001234406.2), SlHXK3 (NP_001234710.1), SlHXK4 (NP_001234717.1), SlHXK5 (XP_004231963.1), SlHXK6 (XP_004251354.1), AtHXK1 (NP_194642.1), AtHXK2 (NP_179576.1), AtHXK3 (NP_175220.2), AtHKL1 (NP_175463.1), AtHKL2 (NP_188639.2), AtHKL3 (NP_195497.1), OsHXK1 (XP_015645221.1), OsHXK2 (XP_015637797.1), OsHXK3 (XP_015621344.1), OsHXK4 (XP_015645316.1), OsHXK5 (XP_015639323.1), OsHXK6 (XP_015618116.1), OsHXK7 (XP_015637554.1), OsHXK8 (XP_015622018.1), OsHXK9 (XP_015614778.1), OsHXK10 (XP_015638932.1), NtHXK1 (NP_001312563.1), NtHXK1a (NP_001312738.1), NtHXK2 (NP_001312431.1), NtHXK3 (NP_001311844.1), NtHXK4a (NP_001312782.1), NtHXK4b (NP_001311993.1), NtHXK5 (AAS60197.1), NtHXK6 (NP_001312380.1), NtHXK7 (AAT77515.1), ZmHXK3a (LOC103650768), ZmHXK3b (LOC103636300), ZmHXK4 (LOC542510), ZmHXK5 (LOC100170246), ZmHXK6 (LOC103651223), ZmHXK7 (LOC100283735), ZmHXK8 (LOC100192075), ZmHXK9 (LOC100279587), and ZmHXK10 (LOC100285932). (**B**). Multiple sequence alignment of HXK protein sequences in tomato. Black and gray show 100% and 75-99% identity on protein sequences, respectively.

To investigate the phylogenetic relationships between SlHXKs and other known HXKs from dicots (6 in *Arabidopsis thaliana* and 9 in *Nicotiana tabacum*) and monocots (10 in *Oryza sativa* and 9 in *Zea mays*), 40 entire HXK protein sequences were used to construct a phylogenetic tree. The accession numbers of the HXK protein sequences were listed in Table S3. The phylogenetic tree was clustered into six different groups (groups I–VI), of which SlHXKs were distributed among four groups (Figure 1A). Members of group I belonged to dicotyledonous plants, which contained transmembrane regions/membrane anchoring domains. Three SlHXKs (SlHXK1-3) were grouped into group I (type B HXKs) and one SlHXK was grouped into group IV, V, and VI. However, no SlHXK was classed into group II (type B HXKs) or III (type C HXKs), and only HXKs from monocotyledons were classed into group II and III. Group IV (type A HXKs) contained SlHXK4, which was closely related to AtHXK3, OsHXK4, and NtHXK2. SlHXK6 belonged to group V (type B HXKs) and was closely related to AtHKL1-2 and NtHXK6. Interestingly, SlHXK5 and AtHKL3 form the same branch, which did not contain any members of tobacco, rice, or maize HXKs. For further characterization of the SlHXK proteins, the DNAMAN software was used to align the protein sequences. The multiple alignments showed that SlHXK1-6 shares 36.3–82.7% similarity (Figure 1B and Table S4). The two phosphate sites, one sugar binding site, one adenosine binding site, one α-helix site, and two connection sites of SlHXK were located in the same highly conservative region (Figure 1B).

### 2.3. Gene Duplication Survey and Collinearity Analysis

To clarify the duplication events among the *SlHXK* genes on chromosome segments, a collinearity analysis was performed using MCScanX program, TBtools (V2.120) software.

**Figure 2:**
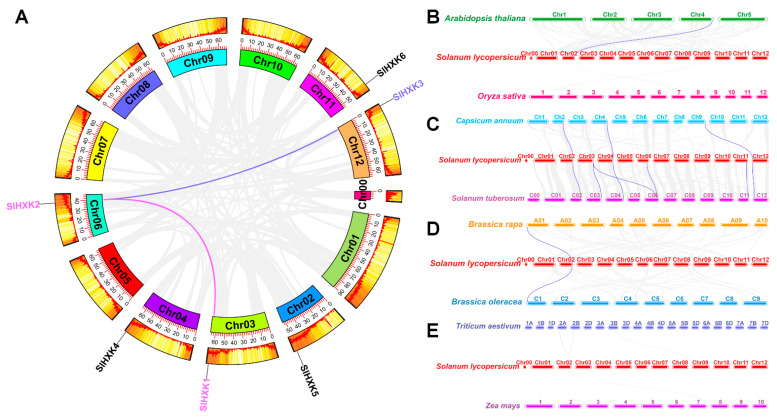
*SlHXK* gene replication and collinearity analysis with the other 8 plant HXK family genes. (**A**). Segmental duplications of *SlHXK* genes in tomato chromosomes. The curves of different colors indicate the collinearity relationships of *SlHXK* genes. The corresponding *SlHXKs* located in segmental duplications are marked with colors, and gene names that have no collinear relationships are marked in black. (**B**–**E**). Synteny analysis of HXK family genes between tomato and eight other plant species: (**B**) *Arabidopsis thaliana* and *Oryza sativa*, (**C**) *Capsicum annuum* and *Solanum tuberosum*, (**D**) *Brassica rapa* and *Brassica oleracea*, (**E**) *Triticum aestivum* and *Zea mays*.

The results showed that two gene pairs with segmental duplications were found, including *SlHXK1–SlHXK2* and *SlHXK2–SlHXK3* (Figure 2A). Interestingly, these linked *SlHXK* genes belonged to the same subgroup in the phylogenetic analysis. In order to further explore the origin and evolutionary relationship of *SlHXK* genes, the six *SlHXK* genes of tomato and the related genes of eight representative species were analyzed through collinearity, including two representative model plants (*Arabidopsis thaliana* and *Oryza sativa*), two solanaceous plants (*Capsicum annuum* and *Solanum tuberosum*), two cereal plants (*Zea mays* and *Triticum aestivum*), and two Brassica plants (*Brassica oleracea* and *Brassica rapa*). The numbers of orthologous genes are one between *Solanum lycopersicum* and *Arabidopsis thaliana*, three between *Solanum lycopersicum* and *Capsicum annuum*, six between *Solanum lycopersicum* and *Solanum tuberosum*, one between *Solanum lycopersicum* and *Brassica oleracea*, and one between *Solanum lycopersicum* and *Brassica rapa* (Figure 2B–E). However, no such related genes were found between *Solanum lycopersicum* and *Oryza sativa* and *Zea mays* and *Triticum aestivum* (Figure 2B,E). Interestingly, we found the highest collinearity between *Solanum lycopersicum SlHXKs* and *Solanum tuberosum* genes, which is because they may belong to Solanaceae and be relatively close (Figure 2C). It is worth mentioning that *SlHXK5* was collinear with the detected orthologous genes of other species, indicating that it may come from the common ancestor of these plants (Figure 2B–D).

### 2.4. Gene Structure, Cis-Element, and Motif Analysis

**Figure 3:**
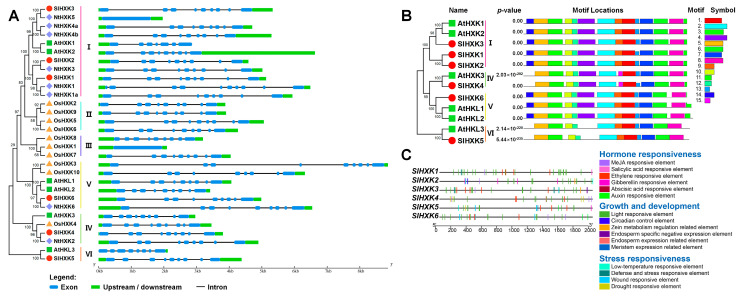
Gene structure, conserved motif and promoter *cis*-acting element analysis of HXK family genes. (**A**). Gene structure of HXK genes in tomato, *Arabidopsis*, tobacco and rice. Blue ovals indicate exons, black lines indicate introns, and green rectangular boxes indicate untranslated regions. (**B**). The motif distribution of HXK proteins in tomato and *Arabidopsis*. Motifs were analyzed using the MEME web server, and 15 conserved motifs were boxed in colors. (**C**). *Cis*-elements in the promoters of *SlHXK* genes. Different colored rectangles represent different *cis*-elements that are potentially involved in stress, growth and development, or hormone regulation.

On the basis of the CDS and genome sequences of the HXK family members, the gene structure was analyzed using the GSDS tool. The results showed that most members of the HXK gene family had 9 exons, except for *OsHXK1* and *NtHXK5* (1 exon), *AtHXK1* (7 exons), *AtHKL3* (8 exons), and *NtHXK1* (10 exons) (Figure 3A). Meanwhile, in tomato, all *SlHXK* genes had nine exons and eight introns (Figure 3A). Therefore, the HXK genes had similar exon–intron structures in different species, indicating the evolutionary conservatism of HXK gene structures in different plant species.

To analyze SlHXK protein motif characteristics, the MEME online tool was used to predict conserved motifs. A total of 15 conserved motifs in SlHXK and AtHXK protein sequences were predicted (Figure 3B and Figure S2). SlHXK1-3, SlHXK6 and AtHXK1-2, AtHKL1-2 had highly similar motif distributions, including motifs 1–14. Both SlHXK4 and AtHXK3 had 13 motifs, except for motifs 9 and 14. In addition, SlHXK5 and AtHKL3 had 11 motifs, of which the N terminal lacked motif 14 and the C terminal lacked motif 11 (Figure 3B). Interestingly, the classification of the four groups in the motif distribution was almost identical to the clustering of the phylogenetic tree.

We further analyzed the *cis*-acting elements in the *SlHXK* promoter regions. The *cis*-regulatory elements in the *SlHXK* promoter regions were divided into three categories, including hormone, growth and development, and abiotic stress response elements. Light-responsive elements were found in promoters of all *SlHXKs* (Figure 3C). Analysis of hormone-response-related elements showed that the number of ethylene (ETH) and methyl jasmonate (MeJA) responsive elements was the highest, followed by abscisic acid (ABA), gibberellin (GA), and auxin (IAA) responsive elements. Except for *SlHXK5*, other promoters of *SlHXKs* all contained the MeJA responsive elements (TGACG-motif and CGTCA-motif). All promoters of *SlHXKs*, except for *SlHXK2*, contained ETH response elements (ERE) (Figure 3C and Table S5). In addition, the *cis*-element involved in salicylic acid (SA) (TCA-element) was found in the promoter of *SlHXK1*. At least two hormone responsive elements were found in promoters of all *SlHXKs*. Many abiotic-stress-related elements, such as low temperature responsive element (LTR), wound responsive element (WUN-motif), drought responsive element (MBS) and defense and stress responsive elements (TC-rich repeats), were also identified from the promoter sequences of *SlHXKs* (Figure 3C).

### 2.5. Expression Patterns of SlHXKs

**Figure 4:**
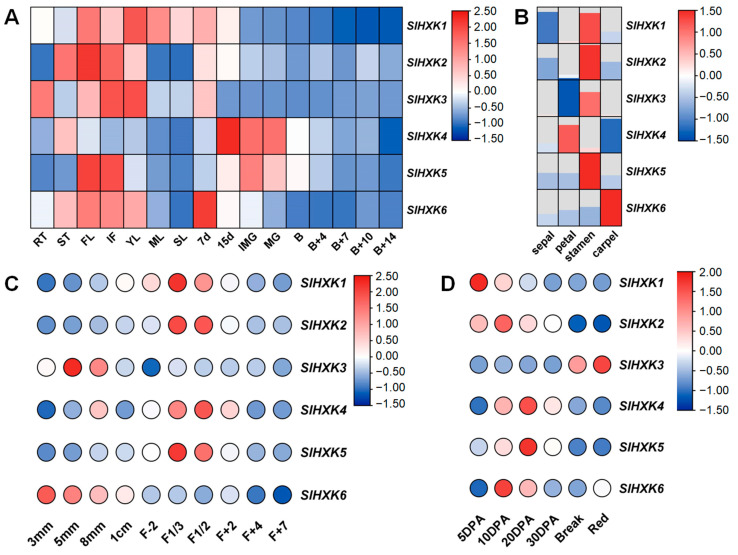
Heat map of the expression patterns of *SlHXK* genes in different tissues of tomato. (**A**). Relative expression levels of *SlHXK* genes in various tissues of wild-type tomato. RT: root; ST: stem; FL: flower; IF: inflorescence; YL: young leaf; ML: mature leaf; SL: senescent leaves; 7 d: fruit 7 d after pollination; 15 d: fruit 15 d after pollination; IMG: immature green fruits; MG: mature green fruits; B: breaker fruits; B + 4: fruits 4 days after breaker; B + 7: fruits 7 days after breaker; B + 10: fruits 10 days after breaker; B + 14: fruits 14 days after breaker. (**B**). Expression patterns of *SlHXK* genes in different floral organs of WT. (**C**). Expression patterns of *SlHXK* genes at different developmental stages of wild-type flowers. 3 mm: 3 mm long flower buds; 5 mm: 5 mm long flower buds; 8 mm: 8 mm long flower buds; 1 cm: 1 cm long flower buds; F-2: 2 d flower before anthesis; F1/3: flowers with 1/3 of petals open; F1/2: flowers with 1/2 of petals open; F+2: flowers 2 d after anthesis; F + 4: flowers 4 d after anthesis; F + 7: flowers 7 d after anthesis. (**D**). Expression patterns of *SlHXK* genes at different developmental stages of seeds. 5 DPA: seeds of pollinated 5 d fruits; 10 DPA: seeds of pollinated 10 d fruits; 20 DPA: seeds of pollinated 20 d fruits; 30 DPA: seeds of pollinated 30 d fruits; Break: seeds of breaker fruits; Red: seeds of red fruits.

To explore the spatial expression pattern of *SlHXKs*, the qRT-PCR was performed to detect their expression levels in different tissues of tomato (root, stem, flower, inflorescence, young leaves, mature leaves, senescent leaves, and fruit at different developmental stages). The results showed that *SlHXKs* were expressed in various tomato tissues, but they had low expression levels after tomato fruit ripening. All genes, except for *SlHXK4*, were highly expressed in flowers and inflorescence. Moreover, *SlHXK1* also had a high expression level in leaves (young leaves, mature leaves, and senescent leaves). In contrast, the expression level of *SlHXK4* was higher mainly at the early stage of fruit development (Figure 4A and Figure S3). Considering the high expression of most *SlHXK* genes in flowers, we analyzed the expression profiles of *SlHXK* genes in the sepal, petal, stamen, and carpel and different flower development stages. The results showed that *SlHXK1*, *SlHXK2*, *SlHXK3,* and *SlHXK5* were highly expressed in stamen, *SlHXK4* was highly expressed in the petal and *SlHXK6* was highly expressed in the carpel (Figure 4B). *SlHXK1*, *SlHXK2*, *SlHXK4,* and *SlHXK5* showed similar expression patterns at different stages of flower development, and their expression levels at the flowering stage (F1/3 and F1/2) were higher than those before and after flowering (Figure 4C). In addition, we analyzed the expression patterns of *SlHXKs* at different stages of seed development [44,45]. The results showed that most of the *SlHXK* genes were highly expressed at the early stage of seed development, except for *SlHXK3* (Figure 4D). In general, the tissue-specific expression of *SlHXKs* indicated that different members may play significant roles in different physiological and developmental processes.

### 2.6. Expression Analysis of SlHXKs Under Various Abiotic Stresses

**Figure 5:**
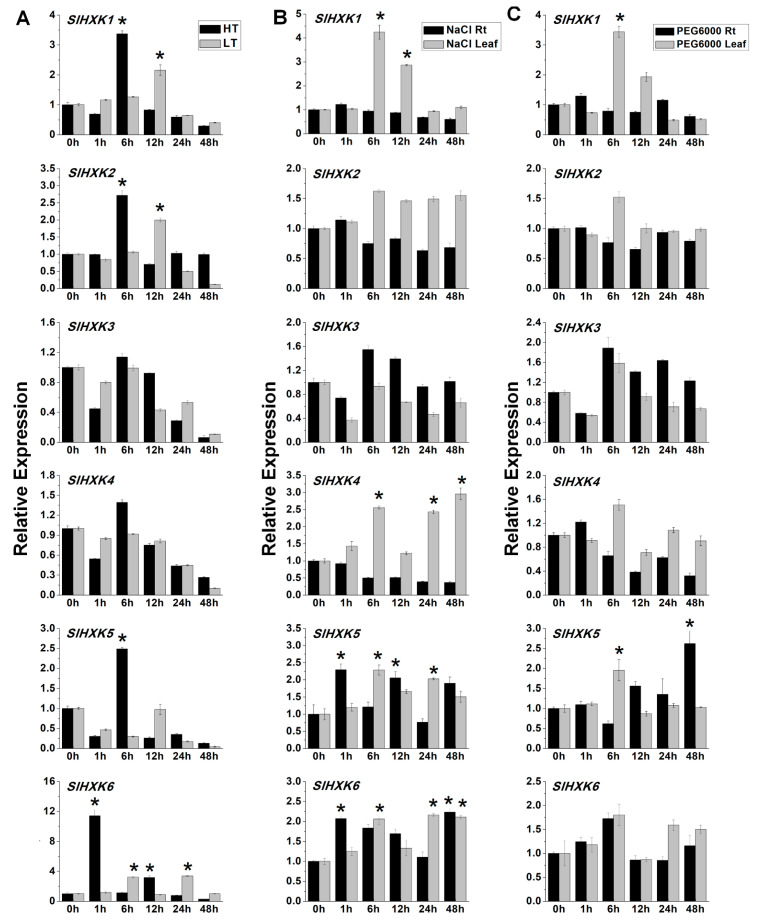
Expression analysis of *SlHXK* genes under abiotic stress treatment. (**A**). Expression analysis of *SlHXK* genes under high- and low-temperature treatments. (**B**). Expression analysis of *SlHXK* genes under NaCl treatment. (**C**). Expression analysis of *SlHXK* genes under PEG6000 treatment. The expression level of 0 h was set to 1, and the dashed line indicates the twofold threshold. *SlEF1α* was used as an internal reference gene. All data represent means (±SE) of three biological replicates. The two-fold expression changes of SlHXK genes in each post treated sample compared to 0h sample is considered to be the significant expression changes. The significant differences were marked with the asterisks between the 0h sample and each post treated sample using Student’s *t*-test: * *p* < 0.05.

Recent studies have shown that HXK genes are involved in plant stress responses [29,46]. To determine whether *SlHXK* genes respond to abiotic stress, the expression levels of *SlHXK* genes under high-temperature (42 °C), low-temperature (4 °C), sodium chloride (NaCl), and PEG6000 treatments were analyzed through qRT-PCR. The transcript levels of four *SlHXK* genes (*SlHXK1*, *SlHXK2*, *SlHXK5*, and *SlHXK6*) were significantly increased after high-temperature treatment. The expression levels of *SlHXK1*, *SlHXK2,* and *SlHXK6* were increased more than two-fold after the low-temperature treatment (Figure 5A). Under salt stress, the expression levels of *SlHXK1* and *SlHXK4* were significantly up-regulated in leaves, and the expression of *SlHXK5* and *SlHXK6* showed a similar trend, while the expression of *SlHXK3* and *SlHXK4* did not change significantly in roots (Figure 5B). The role of *SlHXKs* in drought stress was explored using PEG6000 to simulate drought. In leaves, the expression level of *SlHXK1* reached a peak 6 h after treatment. In roots, the expression level of *SlHXK5* was significantly increased 48 h after treatment. In addition, the expression levels of the remaining four *SlHXK* genes did not change significantly in leaves and roots (Figure 5C). To preliminarily analyze the response and possible function of *SlHXK* genes under hormone treatment, the relative transcript levels of *SlHXK* genes in leaves after SA, ACC, ABA, IAA, GA, MeJA, and ZT treatments were investigated through qRT-PCR. After treatment with SA, ABA, and ACC, only the expression level of the *SlHXK1* gene was significantly increased at 6 h and 12 h, which was about four to eight times higher than the control level. The expression level of *SlHXK3* was significantly decreased 1 h after SA treatment and significantly increased 6 h and 12 h after ACC treatment. The expression levels of *SlHXK5* and *SlHXK6* were significantly increased 1 h after ACC treatment and then decreased to the control level (Figure S4). After GA treatment, the expression level of *SlHXK3* was significantly increased at 6 h and then significantly decreased at 24 h. The expression level of *SlHXK5* was significantly up-regulated only at 48 h, while the expression level of *SlHXK6* was significantly increased at 1 h and 48 h. The expression of *SlHXK1* was significantly down-regulated 24 h after GA treatment. The expression of four *SlHXK* genes (*SlHXK1*, *SlHXK3*, *SlHXK5,* and *SlHXK6*) was significantly suppressed 24 h after IAA treatment. The expression of *SlHXK2* and *SlHXK4* genes was not significantly changed after GA and IAA treatments (Figure S5). After ZT treatment, the expression of the *SlHXK1* gene was significantly up-regulated at 6 h and 12 h, and the expression of the *SlHXK3* gene was significantly up-regulated at 6 h, while the expression of *SlHXK4* and *SlHXK6* was significantly decreased at 48 h. After MeJA treatment, only the expression of *SlHXK2* and *SlHXK6* was significantly up-regulated, while the expression of the *SlHXK3* gene was significantly decreased at 1 h (Figure S6).

### 2.7. Effects of Sugars on SlHXK Genes’ Expression

**Figure 6:**
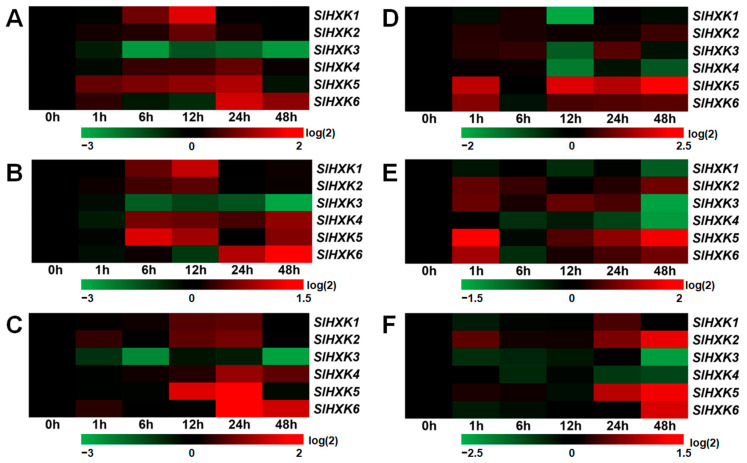
Heat map of *SlHXK* genes’ expression analysis in roots and leaves under glucose, fructose, and sucrose treatment. (**A**) The 3% glucose treatment, leaves. (**B**) The 3% fructose treatment, leaves. (**C**) The 3% sucrose treatment, leaves. (**D**) The 3% glucose treatment, roots. (**E**) The 3% fructose treatment, roots. (**F**) The 3% sucrose treatment, roots. The average of three biological replicates was used, and the data were normalized for generating heat maps. Red represents higher expression levels than the 0 h control and green represents lower expression levels than the 0 h control.

To study the effects of various sugars on the expression of *SlHXK* genes, the expression profiles of *SlHXK* genes were explored under 3% glucose, 3% fructose, and 3% sucrose treatments using qRT-PCR. In leaves, the expression of *SlHXK1* showed a unique expression pattern, which gradually increased to its peak from 6 to 12 h after glucose and fructose treatments and then decreased to the control level (Figure 6A,B). The expression of *SlHXK2* was significantly induced 12 h after glucose and fructose treatments and 12–24 h after sucrose treatment. The expression profiles of *SlHXK3* after glucose, fructose, and sucrose treatments were similar, and its expression was significantly inhibited after treatments. The expression of *SlHXK4* was significantly induced 24 h after glucose treatment, 6–12 h and 48 h after fructose treatment, and 24–48 h after sucrose treatment. The expression of *SlHXK5* was significantly up-regulated 1–24 h after glucose treatment, reached the highest level 6 h after fructose treatment, and reached the highest level 24 h after sucrose treatment. The expression of *SlHXK6* showed a similar expression profile after treatments with glucose, fructose, and sucrose, and its expression was significantly up-regulated at 24–48 h (Figure 6A–C). Figure 6D–F show the expression profiles of *SlHXK* genes in roots treated with glucose, fructose, and sucrose, respectively. The expression of *SlHXK1*, *SlHXK3,* and *SlHXK4* was significantly inhibited at 12 h, while the expression of *SlHXK5* and *SlHXK6* was significantly induced at 1 h and 12–48 h under glucose treatment (Figure 6D). The expression of *SlHXK1*, *SlHXK3,* and *SlHXK4* was significantly down-regulated at 48 h, while the expression of *SlHXK2*, *SlHXK3*, *SlHXK5,* and *SlHXK6* was significantly up-regulated at 1 h under fructose treatment. In addition, the expression profiles of *SlHXK5* and *SlHXK6* were similar, and their expression levels gradually increased from 6 to 12 h after fructose treatment (Figure 6E). The expression of *SlHXK1* was decreased at 1 h and increased 24 h after sucrose treatment. The expression level of *SlHXK2* was significantly increased 1 h and 24–48 h after sucrose treatment. The expression levels of *SlHXK3* and *SlHXK4* showed a downward trend under sucrose treatment, and the expression level of *SlHXK3* reached a significantly low level 48 h after sucrose treatment. In contrast, the expression levels of *SlHXK5* and *SlHXK6* reached a significantly high level 48 h after sucrose treatment (Figure 6F).

### 2.8. Yeast Complementation of SlHXK5 and SlHXK6

**Figure 7:**
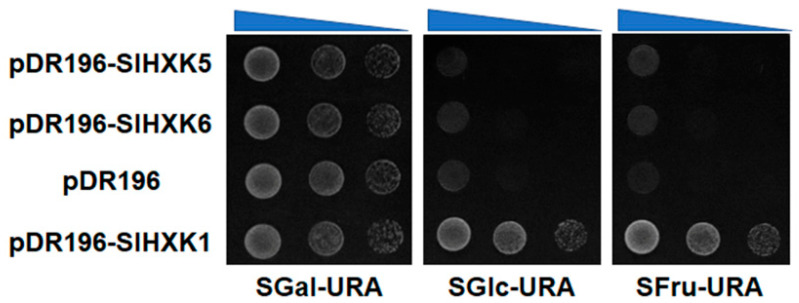
Complementation analysis of HXK-deficient yeast mutants. SGal-URA: lack of uracil and using galactose as the only carbon source; SGlc-URA: lack of uracil and using glucose as the only carbon source; SFru-URA: lack of uracil and using fructose as the only carbon source.

Previous studies have demonstrated that SlHXK1, SlHXK2, SlHXK3, and SlHXK4 have hexose phosphorylation activity [41,43]. To evaluate whether SlHXK5 and SlHXK6 have hexokinase activity, we cloned the ORFs of SlHXK1, SlHXK5, and SlHXK6 into the pDR196 yeast expression vector to generate pDR196-*SlHXK1*, pDR196-*SlHXK5,* and pDR196-*SlHXK6* plasmids. The yeast with transformed pDR196-*SlHXK1* plasmids was used as the positive control, and the yeast with transformed empty pDR196 plasmids was used as the negative control. The results showed that the yeast transformed with empty pDR196, pDR196-*SlHXK1*, pDR196-*SlHXK5,* and pDR196-*SlHXK6* plasmids grew normally on the medium (SGal-URA) with galactose as the sole carbon source. However, only the yeast with transformed pDR196-*SlHXK1* plasmids could grow on the medium with glucose or fructose as the sole carbon source, while the yeast with transformed empty pDR196, pDR196-*SlHXK5,* and pDR196-*SlHXK6* plasmids could not grow normally on these mediums (Figure 7). These results suggested that SlHXK5 and SlHXK6 were unable to phosphorylate glucose and fructose in yeast cells.

## 3. Discussion

### 3.1. Characterization of SlHXKs in Tomato

HXK family proteins play key roles in sugar sensing, sucrose metabolism, catalyzing hexose phosphorylation, providing energy, and regulating plant growth and stress response [19]. Currently, studies on HXK are mainly focused on *Arabidopsis thaliana* and rice, but there are a few reports in tomato. Previous studies have reported four HXK genes in tomato, which is fewer than in other species, such as *Arabidopsis*, tobacco, and rice [6,7,9,41,43]. In the present study, six non-redundant HXK genes (*SlHXK1-6*) were identified from the tomato genome. Among them, the coding regions of *SlHXK1-4* were consistent with those previously reported. Previous studies on gene structure in *Arabidopsis* have shown that all *AtHXKs* contain seven to nine exons and six to eight introns [7]. Similarly, this study found similar gene structures in tomato and three other species with highly similar exon numbers (Figure 3A). High similarity of protein sequences is usually associated with functional conservation. In this study, amino acid sequence alignment results showed that the HXK homolog in tomato contains conserved regions similar to the previously reported HXK, including phosphorylation sites, sugar binding sites, connection sites, α-helices, and adenosine binding sites, which are important for the function of plant HXK (Figure 1B) [47]. Motif analysis showed that the distribution of the 15 motifs predicted in the tomato HXK protein was consistent with that in *Arabidopsis*, and HXK proteins clustered in the same branch had the same motif type and distribution, indicating the conserved evolution of *SlHXK* genes (Figure 3B) [48]. It is well-known that *cis*-elements are essential structures required for downstream transcription [49]. In this study, the plant hormone responsive, light responsive, and abiotic stress responsive related elements were observed in the promoter sequences of tomato HXK family members, suggesting the multifarious involvement of tomato HXK proteins in plant growth and development and tolerance to abiotic stress (Figure 3C).

Members of the HXK family in plants can be classified into HXK proteins and HKL (hexokinase-like) proteins based on their ability to phosphorylate glucose [13]. In *Arabidopsis*, AtHXK1-3 encode HXK proteins with catalytic activity, which can phosphorylate glucose, while AtHKL1-3 encode HKL proteins lacking catalytic activity, which cannot phosphorylate glucose [7]. In tobacco, NtHXK6 lacks glucose phosphorylation activity, also known as NtHKL1 [6]. Phylogenetic analysis showed that SlHXK1-3 clustered in the same branch as AtHXK1-2, SlHXK4 clustered with AtHXK3, NtHXK2, and OsHXK4, SlHXK5 clustered in a separate branch with AtHKL3, and SlHXK6 clustered with AtHKL1, AtHKL2, and NtHXK6 (Figure 1A). Previous studies have shown that SlHXK1-4 belongs to the HXK proteins with catalytic activity [41,42,43]. We hypothesized that SlHXK5 and SlHXK6 are HKL proteins with no catalytic activity. The absence of glucose phosphorylation activity of SlHXK5 and SlHXK6 was further confirmed by yeast mutant complementation experiments (Figure 7). In short, these results demonstrate that SlHXK5 and SlHXK6, which are similar to HKL proteins AtHKL1-3 from *Arabidopsis*, may have regulatory roles in sensing sugar signals but no catalytic activity in sugar metabolism. Although these proteins lack catalytic activity in sugar metabolism, they can still play roles in sensing sugar signals by acting as sugar receptors.

### 3.2. Evolutionary Patterns Among HXK Genes

Genome replication events are the key driving force for the evolution and expansion of many gene families in plants, which can promote the emergence of new functional genes and species so that plants can better withstand adverse environmental conditions during evolution [50]. In our study, the *SlHXK* genes that showed tandem and segmental duplication events were located in the same subgroup in the phylogenetic tree. In addition, the highest number of homologous genes was found between tomato and potato, which supports the close evolutionary relationship between them. No homologous gene pairs were found between tomato and cereal plants (wheat and maize), which may be due to their genomes undergoing large-scale chromosome rearrangement and fusion, and the loss of selective genes seriously obscured the recognition of collinearity relationships (Figure 2).

### 3.3. Subcellular Localization

Generally, HXKs in plants can be classified into two major types (type A and B) and two minor types (type C and D) based on their N-terminal amino acid sequences [19]. So far, type D is present only in mosses, and type C is present in mosses and monocots, such as rice and maize, without signal peptides and membrane-anchored domains, localized in the cytoplasm [9,26]. However, there are only two types (type A and B) of HXKs in dicotyledons. The N-terminal of type A HXKs contains a chloroplast signal peptide and is located in the chloroplast. The N-terminal of type B HXKs has a membrane-anchored domain and is localized in the mitochondria [7]. In this study, based on the presence of a chloroplast signal peptide or transmembrane structural domain (Table 1) and the previously reported classification, SlHXK1-3 and SlHXK5-6 belong to type B HXKs, and SlHXK4 belongs to type A HXKs, the same type as the other HXKs in the same subgroup of the phylogenetic tree (Figure 1A). It has been previously confirmed that SlHXK1-3 is localized in mitochondria and SlHXK4 is located in chloroplasts based on GFP fusion protein assays [51]. The prediction results of subcellular localization showed that SlHXK5 and SlHXK6 were located in mitochondria, indicating the close correlation between the subcellular localization of HXK protein and their N-terminal sequences. However, their accurate subcellular localization in cells still needs to be confirmed through further experiments.

### 3.4. Expression Profiling and Functional Prediction of SlHXKs in Tomato

Tissue-specific expression patterns can provide a better understanding of the functions of plant genes [52]. In *Arabidopsis*, most *AtHXK* genes are widely expressed in all tissues, while *AtHKL3* is mainly expressed in flowers [7]. In rice, the *OsHXK* genes show a similarly broad expression profile, except for *OsHXK1*, which is not detected in any tissues, and *OsHXK10*, which is only expressed in flowers [9]. In our study, *SlHXKs* were expressed in different tissues, but their expression levels were extremely low after fruit ripening in tomato. In addition, the *SlHXK* genes showed different expression patterns in different tissues of tomato, indicating the tissue specificity of *SlHXK* gene expression. Analysis of *SlHXK* gene expression levels in four-wheel flower organs showed that *SlHXK1-3* and *SlHXK5* were highly expressed in stamens, *SlHXK4* was highly expressed in petals, and *SlHXK6* was highly expressed in carpels. *SlHXK1-2* and *SlHXK4-5* exhibited similar expression patterns at different stages of flower development. Except for *SlHXK3*, the expression levels of most *SlHXK* genes were higher in the early stages of seed development (Figure 4). These results suggest that the roles of *SlHXKs* in different tissues and developmental stages may be different.

Hexokinase not only plays important roles in plant growth and development but also participates in plants’ response to abiotic stresses [17]. In *Arabidopsis thaliana*, overexpression of *AtHXK1* results in stomatal closure, reduced transpiration, and enhanced drought tolerance of the plant [53]. Under osmotic, salt stress, or low-temperature conditions, the expression of *AtHXK2* is significantly induced, while the expression of *AtHXK3* and *AtHKL1* is significantly inhibited [30,31]. The expression of *AtHKL3* is significantly induced under salt, drought, and high- and low-temperature stresses [30]. In addition, many cis-acting elements related to stress resistance have been identified in the promoter region of the *MdHXK1* gene in apple, and its expression can be induced by NaCl and low temperatures [7]. Overexpression of *MdHXK1* enhances Na^+^/H^+^ transport activity and improves the tolerance of apple plants to salt stress [54]. However, little is currently known about the role of *SlHXKs* in stress tolerance in tomato. Identification of gene expression will help to accelerate the determination of gene function. In this study, the expression levels of *SlHXK* genes were examined through qRT-PCR under various stress (high temperature, low temperature, salt, and drought) treatments. The results showed that most of the *SlHXKs* detected were significantly and differentially induced under various abiotic stresses. In particular, the expression level of *SlHXK1* was significantly up-regulated after high-temperature, low-temperature, salt, and drought treatment, suggesting that this gene may contribute to the reduction of damage caused by adverse stresses (Figure 5).

Glucose is the primary carbon and energy source for most organisms, and it is the most studied sugar in plants. HXK is not only the first glucose sensor found in plants but also the only enzyme in plants that can phosphorylate glucose. HXK catalyzes the first step of glycolysis by phosphorylating glucose to glucose-6-phosphate, which provides energy and a number of important intermediate metabolites for plant growth and development [4,5]. Glucose and its metabolites can also affect plant development by affecting the transcription of some genes through HXK-dependent phosphorylation functions or metabolic sensing pathways [19]. Furthermore, it has been shown that in addition to its catalytic activity, HXK acts as a glucose sensor to regulate gene expression and secondary metabolism through HXK-dependent signaling pathways [21,33]. Numerous studies have shown that the glucose signaling pathway mediated by HXK is closely related to a variety of plant hormones, and its signal network is crucial for plant growth and development [32,33]. It has been shown that glucose promotes the degradation of EIN3, a key transcription factor of the ethylene signal transduction pathway, through a pathway dependent on AtHXK1 [35,55]. AtHXK1 also regulates the inhibitory effect of glucose on the ethylene response factor ERF1 [56]. In addition, the addition of exogenous ethylene synthesis precursor ACC (1-aminocyclopropenyl-1-carboxylic acid) can relieve the inhibition of glucose on the development arrest of *Arabidopsis* seedlings, which requires the participation of the AtHKL1 protein [39,57]. ABA is involved in AtHXK1-mediated stomatal closure in guard cells [19]. A recent study found that HXK1 signaling interacts with the IAA, strigolactone, and cytokinin pathways to promote shoot branching and regulates plant architecture in rose, pea, and *Arabidopsis thaliana* [58]. In short, there are complex interactions between HXK-mediated sugar signaling and various hormones, and the signaling network formed by HXK is crucial for plant growth and development. At least two or more elements related to hormone responsive were found in the promoter of *SlHXKs* (Figure 3C). In order to determine the possible functions of *SlHXK* genes under hormone stress, the expression levels of *SlHXK* genes under various hormone (SA, ABA, ACC, GA, IAA, ZT, and MeJA) treatments were examined through qRT-PCR in this study. The results showed that most *SlHXKs* exhibited significantly differential expression under various hormone treatments, suggesting the critical and diverse functions of *SlHXK* genes in response to hormones. The expression of *SlHXK1* was significantly induced after ACC, ABA, SA, and ZT treatments. The expression of *SlHXK2* was only significantly induced by MeJA. *SlHXK3*, *SlHXK5,* and *SlHXK6* were significantly induced by ACC and GA (Figures S4–S6). These results suggest that *SlHXK* genes may play important roles in hormone response. In this study, the expression levels of *SlHXK* genes were further examined through qRT-PCR under 3% glucose, 3% fructose, and 3% sucrose treatments. Compared with the control, *SlHXK* genes showed varying degrees of differential expression after different sugar treatments (Figure 6). The combination of *cis*-acting elements and gene expression analysis showed that SlHXKs crosstalk with sugar, plant hormone, and stress tolerance processes.

At present, it has been reported that HKL proteins exist in various species, such as *Arabidopsis thaliana*, rice, and tobacco, but their lack of catalytic activity makes it challenging to determine their functions. AtHKL1 has no catalytic function, but it is involved in the regulation of plant growth and development, and it is a negative regulator of growth in *Arabidopsis thaliana*, affecting seedling growth in response to glc and auxin [57]. The loss of function of *OsHXK3*, a gene encoding a hexokinase-like (HKL) protein, plays a key role in controlling the grain size of rice [40]. Sugar signaling plays an important role in plants’ response to stress, including heat, cold, drought, and salt stress [59]. HKL proteins can affect plants’ responses to these stresses by participating in the sugar signaling pathway [39]. Although HKL proteins cannot phosphorylate glucose and fructose, they may still play important roles in plants’ development and response to environmental and biological stress. Similarly, we found that SlHXK5 and SlHXK6 could not phosphorylate glucose and fructose, but their expression levels were significantly up-regulated after abiotic stress treatments, such as high temperatures, salt, and drought. The functions of non-catalytic proteins SlHXK5 and SlHXK6 are still unclear and deserve further investigation. *SlHXK* genes may play different key roles in various signaling cascades through a potentially complex network of interactions. Elucidating the function of non-catalytic proteins is an ongoing challenge for researchers. Our research provides some theoretical basis for future exploration of the functions of SlHXK5 and SlHXK6 in tomato.

## 4. Materials and Methods

### 4.1. Plant Materials and Treatments

In our study, the tomatoes (*Solanum lycopersicum*, Mill. cv. Ailsa Craig, AC^++^) were planted in the greenhouse with 16 h of daylight (25 °C) and 8 h of darkness (18 °C), 250 µmol m^−2^ s^−1^ light intensity, and 80% relative humidity. The tomato flowers were tagged at anthesis. The days after anthesis were used to distinguish the developmental period of tomato fruits. The flowers, fruits, roots, stems, seeds, and leaves were selected to analyze the expression profiles. Healthy seedlings (about 4 weeks old) with consistent growth were selected for abiotic stresses and hormone treatments. Salt and drought stress were performed by soaking tomato roots in NaCl (300 mM) and PEG6000 (20%), and then the roots and leaves were harvested, respectively. Heat and cold stresses were applied by transferring seedlings into 42 °C and 4 °C conditions, respectively, and then the leaves were harvested. For hormone treatments, seedlings were sprayed with 0.1 mM ACC, IAA, MeJA, ZT, ABA, GA, and ETH or 1 mM SA solutions, and then the leaves were collected. Sugar treatments were performed by soaking tomato roots in 3% glucose, 3% fructose, and 3% sucrose, and then the roots and leaves were harvested. All fresh samples were harvested 1, 6, 12, 24, and 48 h after treatment, while the leaves and roots of untreated seedlings were collected as controls. Three independent biological replicates were performed in each treatment. All fresh materials were collected and frozen in liquid nitrogen and then placed at −80 °C for standby.

### 4.2. Quantitative Real-Time PCR (qRT-PCR)

Total RNA was isolated from the samples by using RNA Extraction Kits (TianGen, Beijing, China) according to the manufacturers’ instructions. The cDNA was synthesized by using the PrimeScript reverse transcriptase with gDNA removal (TransGen, Beijing, China) and used for qRT-PCR. And, the qRT-PCR was conducted using SYBR Premix Go Taq on the CFX96^TM^ Real-Time System (Bio-Rad, USA). All reactions were carried out as described before [37]. All qRT-PCR primers are presented in Table S1.

### 4.3. Identification of HXKs in Tomato

To identify all of the possible HXK genes in tomato, the *Arabidopsis* HXK protein sequences (TAIR; http://www.arabidopsis.org/, accessed on 16 November 2022) were used as queries for BLASTP searches of the tomato database. After removing the redundant sequences, a total of 6 candidate genes were obtained. The candidate genes were submitted to the SMART (http://smart.embl-heidelberg.de/, accessed on 24 April 2023), and the Conserved Domain Database (https://www.ncbi.nlm.nih.gov/Structure/cdd/cdd.shtml, accessed on 24 April 2023) was used to confirm the presence of HXK domains of each candidate HXK protein. The physical and chemical features of the SlHXKs, including the molecular weight (MW), isoelectric point (pI), hydrophilic–hydrophobic amino acids, and instability index, were predicted using the online ExPASy tool (https://web.expasy.org/protparam/, accessed on 24 April 2023). SignalP 3.0 (http://www.cbs.dtu.dk/services/SignalP-3.0/, accessed on 24 April 2023) was used to predict the signal peptide. Transmembrane helices (TMHs) were predicted using the TMHMM server (v2.0) (http://www.cbs.dtu.dk/services/TMHMM-2.0/, accessed on 24 April 2023).

### 4.4. Chromosomal Location and Synteny Analysis of HXK Genes

The location information of six *SlHXK* genes on the chromosome was determined according to the tomato GFF annotation file obtained from the NCBI database (https://www.ncbi.nlm.nih.gov/genome/) [60]. The genome sequences and annotations of *Solanum lycopersicum*, *Oryza sativa*, *Arabidopsis thaliana*, *Solanum tuberosum*, *Capsicum annuum*, *Brassica oleracea*, *Brassica rapa*, *Zea mays*, and *Triticum aestivum* were downloaded from NCBI, TAIR, and Ensembl Plants (https://plants.ensembl.org/index.html) to analyze the collinearity of *SlHXK* and genes from other species. The collinearity relationships and gene duplications were obtained using MCScanX (Multiple Collinearity Scan toolkit), and the results were visualized using TBtools [60,61].

### 4.5. Sequence Alignment and Phylogeny of HXK Proteins

For sequence alignments, DNAMAN software (Version 6.0) was used to analyze the amino acid sequences of SlHXKs [62]. The HXK protein sequences of tomato, *Arabidopsis*, tobacco, rice, and maize were aligned using CluastW, and MEGA7 software was used to construct the phylogenetic tree using the neighbor-joining (NJ) method [63]. Furthermore, the pairwise deletion and p-distance option parameters were selected, and the bootstrap value was set to 1000 [63].

### 4.6. Gene Structure, Conserved Motif, and Cis-Element Analyses

The intron–exon structures of *SlHXK* genes were illustrated using the gene structure display server 2.0 (GSDS) (http://gsds.gao-lab.org/) [64]. MEME online software (https://meme-suite.org/meme/tools/meme) was used to analyze the conserved motifs in amino acid sequences [65]. The promoter sequence 2000 bp upstream from the start codon was extracted from the tomato genome file using TBtools. Then, the plantCARE database (http://bioinformatics.psb.ugent.be/webtools/plantcare/html/) was used to predict *cis*-elements in the promoter sequence [66]. Finally, TBtools was used to visualize the *cis*-elements on the promoter sequence [60].

### 4.7. Yeast Complementation Assay

The yeast triple mutant (YSH7.4-3C) is lacking in GLK1, HXK1, and HXK2, and it was kindly provided by Professor Xinchao Wang [14]. The pDR196 vector containing a selective marker URA3 was used as a yeast shuttle vector for transformation [14]. The fragments of *SlHXK1*, *SlHXK5*, and *SlHXK6* were inserted into the pDR196 vector by the *Eco*R I/*Xho* I restriction site. The generated plasmids of pDR196-*SlHXK1*, pDR196-*SlHXK5*, and pDR196-*SlHXK6* were verified through sequencing, and then they were transferred into YSH7.4-3C. The transformed colonies grew on the plate of SGal-URA medium (with galactose as the carbon source, without uracil). Subsequently, the yeasts containing recombinant plasmid and empty plasmid (pDR196) were diluted 10 times and 100 times, respectively. And, 5 μL of each dilution was grown in SGal-URA, SGlc-URA (with glucose as the carbon source), and SFru-URA (with fructose as the carbon source) plates. Yeast cells transformed with pDR196 plasmid were used as the negative control, and yeast cells transformed with pDR196-*SlHXK1* recombinant plasmid were used as the positive control. All needed primers are presented in Table S2.

### 4.8. Statistical Analysis

All data are expressed as the mean ± standard deviation of three independent biological replicates. OriginPro 8 software was used to analyze all data, and the significant difference among the samples was analyzed through Student’s *t*-test (*p* < 0.05).

## 5. Conclusions

In this study, six HXK genes were identified and characterized from the tomato genome. Among these HXK genes, new members that had not been identified from previous studies of the HXK gene family were found in tomato, named *SlHXK5* and *SlHXK6*. Meanwhile, the molecular characteristics, chromosome distribution, phylogenetic relationships, promoter *cis*-acting elements, gene structure, and collinearity of six *SlHXK* genes were systematically analyzed. *SlHXK* genes showed diverse and different expression profiles in different tomato tissues and under different abiotic stress, hormone, and sugar treatments. And, *SlHXK* genes showed obvious and distinct inductions under various abiotic stresses. In particular, the expression of *SlHXK1* was significantly up-regulated following high-temperature, low-temperature, salt, and drought treatments. The inability of SlHXK5 and SlHXK6 to phosphorylate glucose and fructose was confirmed through yeast mutant complementation experiments. These results provide valuable resources for better understanding the biological functions of the tomato HXK genes and provide promising candidates for genetic engineering to enhance tomato stress resistance. Further investigations of the regulatory mechanisms and specific roles of *SlHXK* genes in response to stress tolerance are necessary.

## Figures and Tables

**Table 1:** Physicochemical properties of HXK in tomato.

Gene Name	SlHXK1	SlHXK2	SlHXK3	SlHXK4	SlHXK5	SlHXK6
Gene ID	543,779	543,638	778,210	778,211	101,249,034	101,256,649
Gene length (bp)	5129	4588	5331	3808	4381	4982
mRNA length (bp)	1862	1908	2163	1765	2329	2328
ORF length (bp)	1497	1491	1500	1500	1476	1536
Amino acid length (aa)	498	496	499	499	491	511
MW (kDa)	54.04	53.75	54.19	53.99	54.05	55.50
Isoelectric point	5.91	6.26	5.71	5.52	5.69	5.86
Exon number	9	9	9	9	9	9
Subcellular location	Mitochondrion	Mitochondrion	Mitochondrion	Chloroplast	Mitochondrion	Mitochondrion
Total number of atoms	7623	7594	7682	7626	7629	7850
Instability index	32.23	27.83	26.76	40.5	45.61	53.76
Protein stability	Stable	Stable	Stable	Unstable	Unstable	Unstable
Hydrophilic–hydrophobic amino acids	Hydropathicity	Hydropathicity	Hydropathicity	Hydropathicity	Hydropathicity	Hydropathicity
Transmembrane domain	7–24	7–24	5–24	No	5–27	5–24
Transit peptide	No	No	No	1–31	No	No
Number of TMHs	1	1	1	0	1	1

## Data Availability

All data supporting the findings of this study are available within the paper and within its Supplementary Materials published online.

## Supplementary Materials

The following supporting information can be downloaded at https://www.mdpi.com/article/10.3390/plants14030441/s1, Figure S1: Chromosomal distribution of *SlHXK* genes in tomato genome. Figure S2: The amino acid logos of respective motifs. Figure S3: Heat map of *SlHXK* gene expression in different tissues. Figure S4: Expression analysis of *SlHXK* genes under ACC, SA, and ABA treatments. Figure S5: Expression analysis of *SlHXK* genes under GA and IAA treatments. Figure S6. Expression analysis of *SlHXK* genes under ZT and MeJA treatments. Table S1: Specific primer sequences used for qRT-PCR analysis. Table S2: Details of primer sequences used for yeast complementation assay. Table S3: Accession numbers of HXK genes in *Arabidopsis thaliana*, *Solanum lycopersicum*, *Nicotiana tabacum*, *Oryza sativa,* and *Zea mays*. Table S4: The alignment of each two HXK protein sequences in tomato. Table S5: *Cis*-elements associated with growth and development and hormone and abiotic stress within the *SlHXK* gene promoters.
