# Supplementary material for: Genome-Wide Identification and Expression Analysis of Hexokinase Gene Family Under Abiotic Stress in Tomato
Source: Plants (Basel). 2025 Feb 3;14(3):441. doi: 10.3390/plants14030441 (PMC11819920; doi:10.3390/plants14030441)
Supplement: Supplementary file 1 [file plants-14-00441-s001.zip › Supplementary Information.pdf]

Supplementary Information

Genome-Wide Identification and Expression Analysis of Hexokinase Gene Family Under Abiotic Stress in Tomato

Jing Li [1,2], Xiong Yao [2], Jianling Zhang [3], Maoyu Li [4], Qiaoli Xie [1], Yingwu Yang [1], Guoping Chen [1], Xianwei Zhang [2,*] and Zongli Hu [1,*]

1 Laboratory of Molecular Biology of Tomato, Bioengineering College, Chongqing University, Room 523-1, Campus B, 174 Shapingba Main Street, Chongqing 400030, China; micy180605@163.com (J.L.); qiaolixie@cqu.edu.cn (Q.X.); yangyinwu@cqu.edu.cn (Y.Y.); chenguoping@cqu.edu.cn (G.C.)

2 Chongqing Academy of Agricultural Sciences, Chongqing 401329, China; yuyourice@cqaas.cn

3 Laboratory of Plant Germplasm Resources Innovation and Utilization, College of Agriculture and Biology, Liaocheng University, Liaocheng 252000, China; zhangjianling0520@126.com

4 Chongqing Seed Station, Chongqing 401121, China; m18523046893_1@163.com

* Correspondence: ycty2006@126.com (X.Z.); huzongli71@163.com (Z.H.); Tel.: +86-13996265017 (Z.H.); Fax: +86-2365102507 (Z.H.)

Supplementary Tables

Table S1. Specific primer sequences used for qRT-PCR analysis.

Primer Name	Primer Sequence (5'–3')
q-*CAC*	CCTCCGTTGTGATGTAACTGG
	ATTGGTGGAAAGTAACATCATCG
q-*EF1α*	TACTGGTGGTTTTGAAGCTG
	AACTTCCTTCACGATTTCATCATA
q-*SlHXK1*	ATGGTCCTGTGCCAAAATCTG
	GAAGTCATCTTCTCAAATATCTGTTCAC
q-*SlHXK2*	TGGTAGATTCACGGATAAGGATGTA
	GACCTAAAGTTACCCCATTCCATAT
q-*SlHXK3*	TTCGGTGATTATGTCCCATCC
	CACGAGAGGTGATAATATCGCAC
q-*SlHXK4*	GTCCTCTATGATGTAGCTGGGGTAA
	ATAGTGCTCATATAAGCCTCCATCC
q-*SlHXK5*	ATGGAGAAGCACAGTGTTGATAAG
	CTGCATTAGTGCCCATTCCTAG
q-*SlHXK6*	ATTAGCAGTTCCTTTCGTCTTG
	AGGATTTTGGCTACTTCACTCA

Table S2. Details of primer sequences used for yeast complementation assay.

Primer Name	Primer Sequence (5'–3')
196F	CTCTTTTATACACACATTCA
196R	CTGGCGAAGAAGTCCAAAGC
SlHXK1(196)-F	CCGGAATTCATGAAGAAAGTGACGGTGGGA
SlHXK1(196)-R	CCGCTCGAGCTAAGCATCTTGATCTTCAAGGTACA
SlHXK5(196)-F	CCGGAATTCATGAAGAAGGATGTGGTGGTTT
SlHXK5(196)-R	CCGCTCGAGTTATGGTTGTGAAGCGGCA
SlHXK6(196)-F	CCGGAATTCATGGGGAGGTTAGGAGTTGGG
SlHXK6(196)-R	CCGCTCGAGTCACTGCAGCTGTACTGTATCCG

Table S3. Accession numbers of HXK genes in *Arabidopsis thaliana*, *Solanum lycopersicum*, *Nicotiana tabacum*, *Oryza sativa* and *Zea mays*.

Table S4. The Alignment of each two HXK protein sequences in tomato.

Gene name / Similarity (%)	SlHXK1	SlHXK2	SlHXK3	SlHXK4	SlHXK5	SlHXK6
SlHXK1	100					
SlHXK2	82.7	100				
SlHXK3	70.4	68.1	100			
SlHXK4	53.5	53.5	55.4	100		
SlHXK5	44.6	43.5	45.4	40.8	100	
SlHXK6	53.7	52.7	56.5	50.0	36.3	100

Table S5. *Cis*-elements associated with growth and development, hormone and abiotic stress within the *SlHXK* gene promoters.

Supplementary Figures

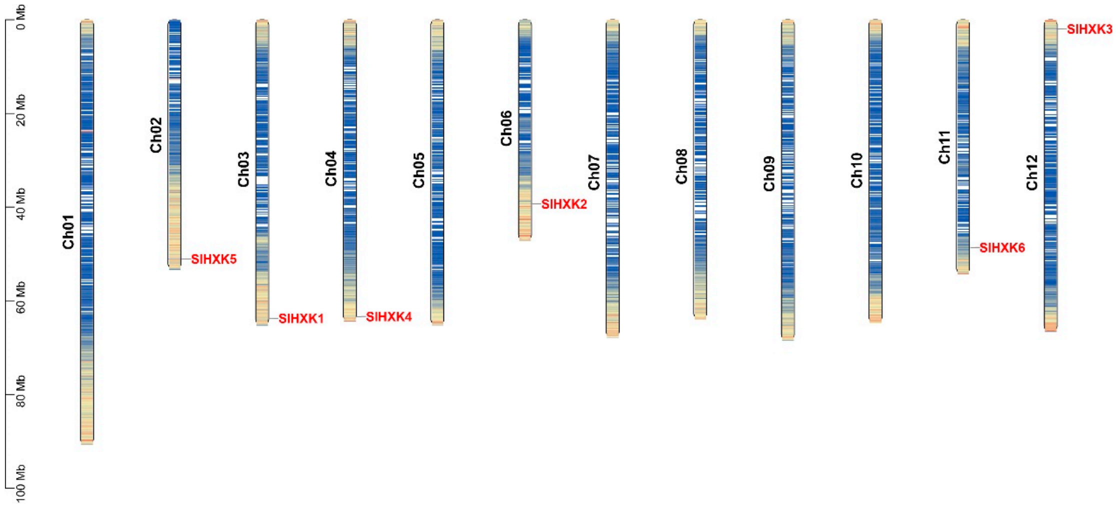

Figure S1. Chromosomal distribution of *SlHXKs* genes in tomato genome.

Motif 1

Motif 2

Motif 3

Motif 4

Motif 5

Motif 6

Motif 7

Motif 8

Motif 9

Motif 10

Motif 11

Motif 12

Motif 13

Motif 14

Motif 15

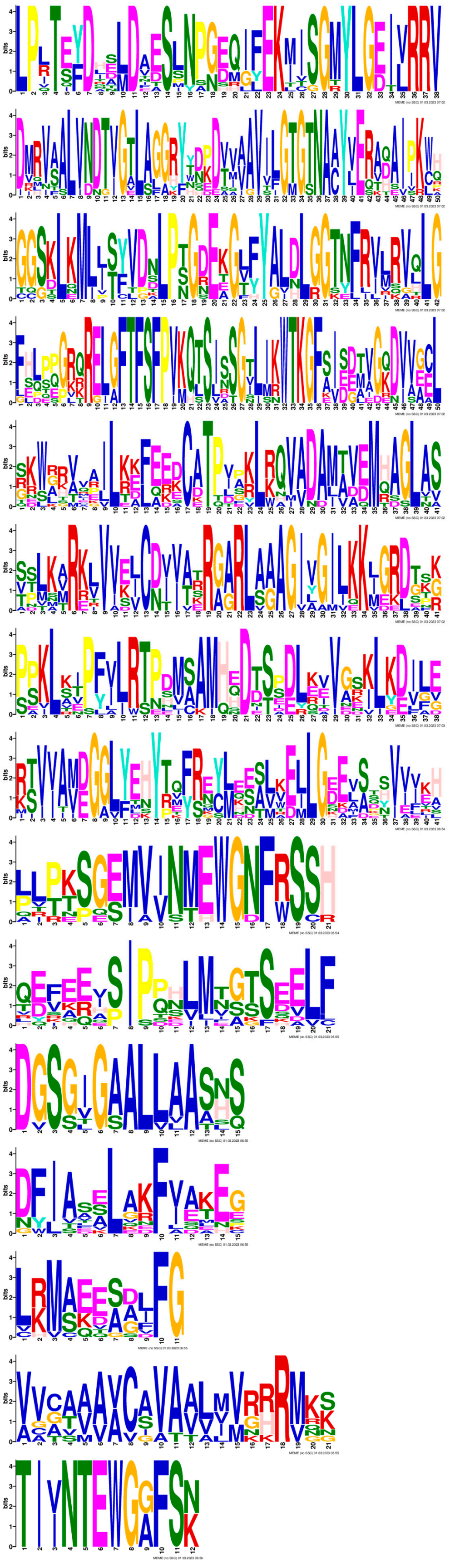

Figure S2. The amino acid logos of respective motifs.

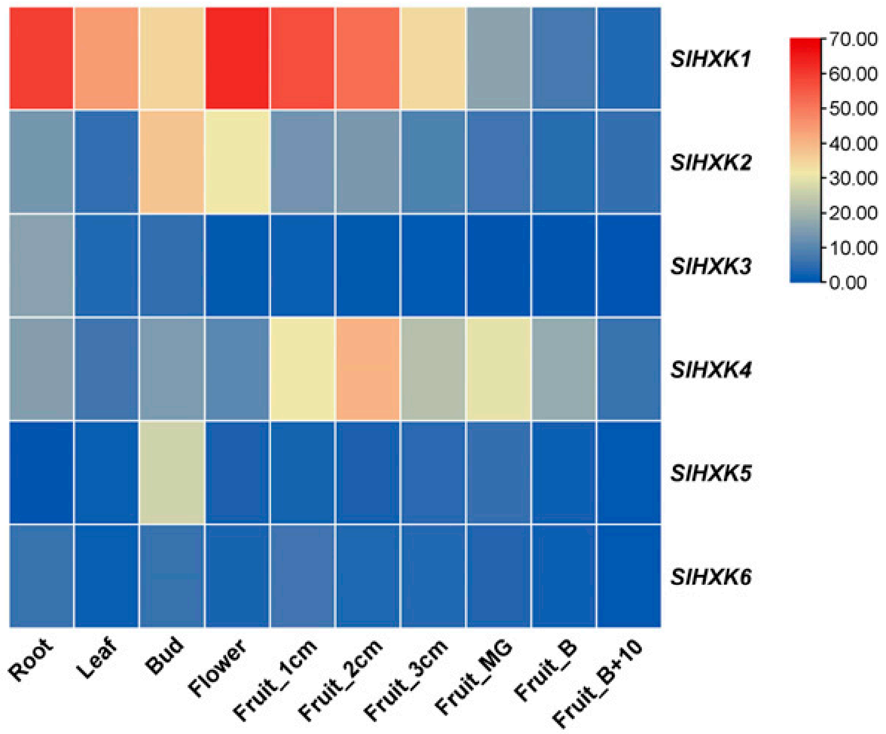

Figure S3. Heat map of *SlHXKs* gene expression in different tissues.

Analysis of *SlHXKs* gene expression levels in tomato based on transcriptome data. Each column represents different tissues of tomato at different developmental stages. The upper right bar indicates the expression level data from high to low (from red to blue).

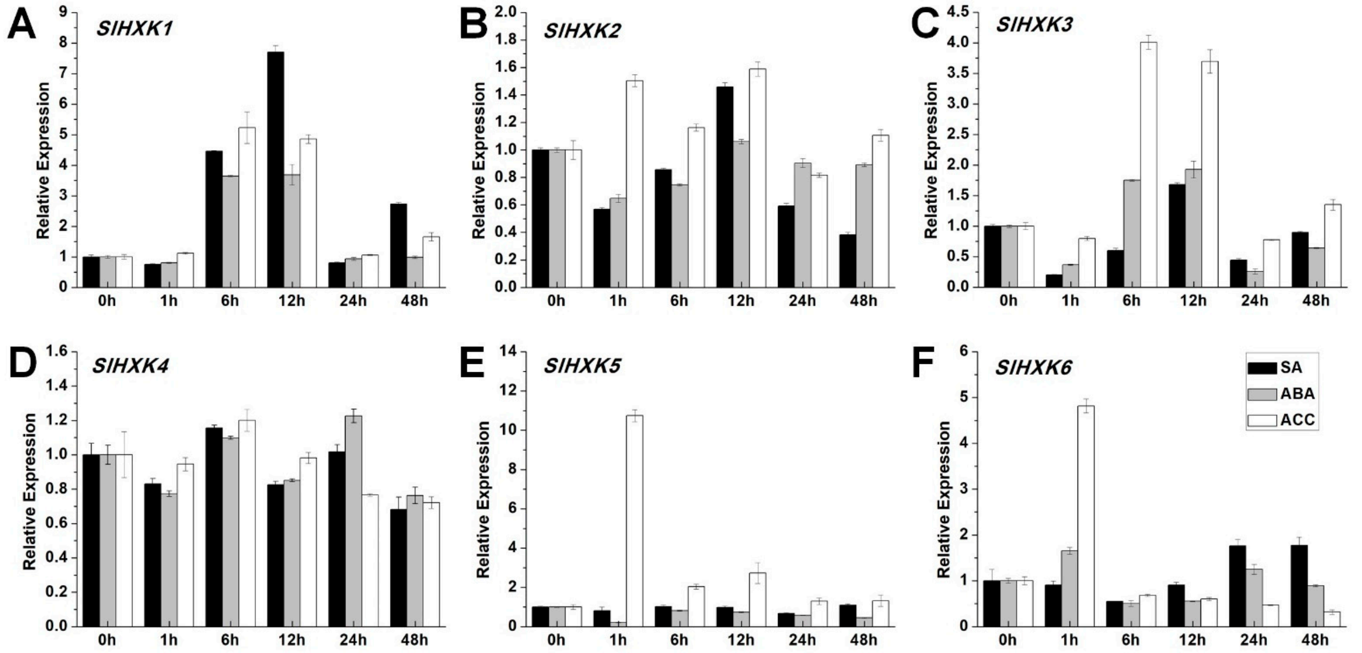

Figure S4. Expression analysis of *SlHXKs* genes under ACC, SA, and ABA treatments.

(A-F). Expression analysis of *SlHXKs* genes under l-aminocyclopropane-l-carboxylic acid (ACC), salicylic acid (SA) and abscisic acid (ABA) treatments. The expression level of 0 h was set to 1. *SlEF1α* was used as an internal reference gene. All data represents means (±SE) of three biological replicates.

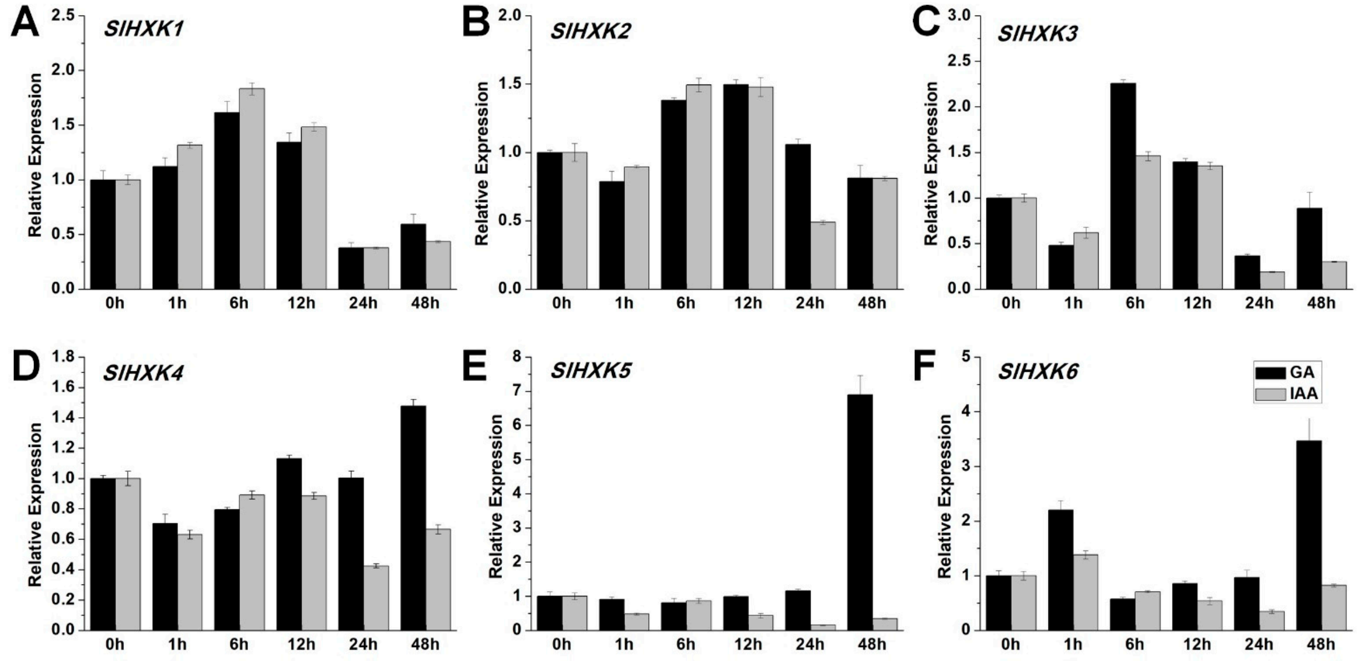

Figure S5. Expression analysis of *SlHXKs* genes under GA and IAA treatments.

(A-F). Expression analysis of *SlHXKs* genes under gibberellin (GA) and auxin (IAA) treatment. The expression level of 0 h was set to 1. *SlEF1α* was used as an internal reference gene. All data represents means (±SE) of three biological replicates.

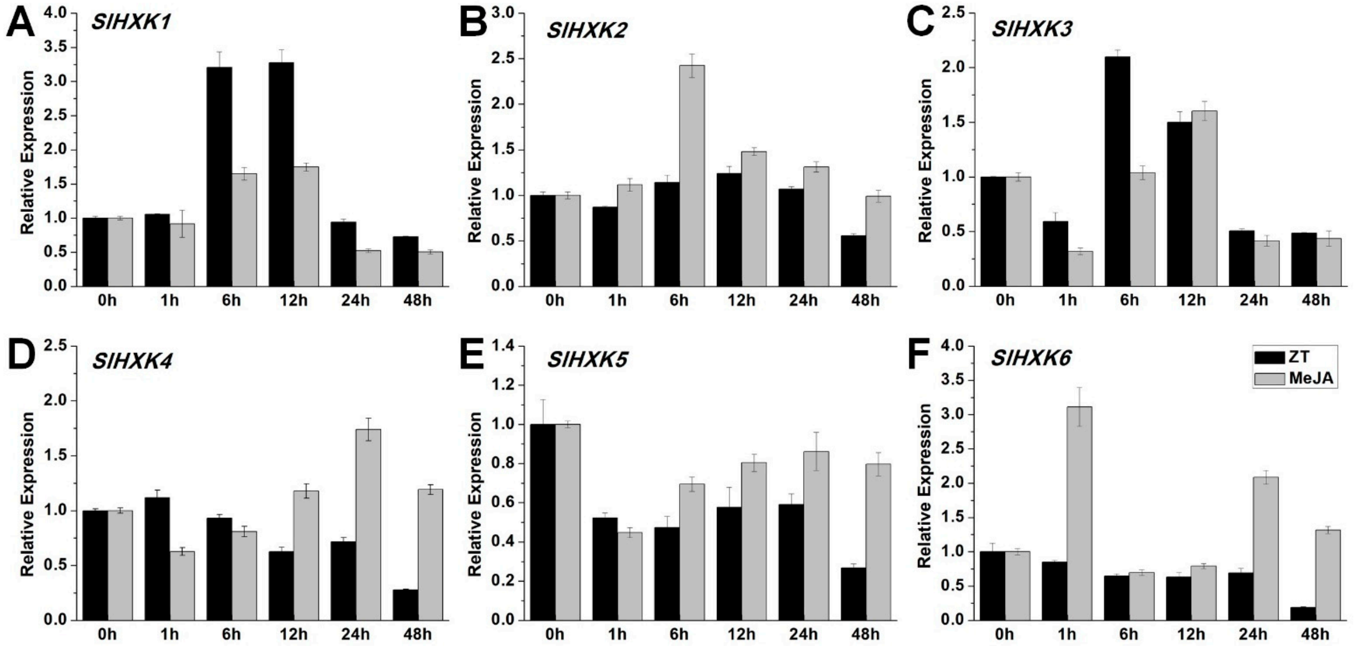

Figure S6. Expression analysis of *SlHXKs* genes under ZT and MeJA treatments.

(A-F). Expression analysis of *SlHXKs* genes under zeatin (ZT) and methyl jasmonate (MeJA) treatments. The expression level of 0 h was set to 1. *SlEF1α* was used as an internal reference gene. All data represents means (±SE) of three biological replicates.
